# Iatrogenic Ocular Surface Diseases Occurring during and/or after Different Treatments for Ocular Tumours

**DOI:** 10.3390/cancers13081933

**Published:** 2021-04-16

**Authors:** Giuseppe Giannaccare, Federico Bernabei, Martina Angi, Marco Pellegrini, Antonio Maestri, Vito Romano, Vincenzo Scorcia, Pierre-Räphael Rothschild

**Affiliations:** 1 Department of Ophthalmology, University Magna Græcia of Catanzaro, Viale Europa, 88100 Catanzaro, Italy; vscorcia@libero.it; 2 Service d’ophtalmologie, Ophtalmopôle de Paris, Hôpital Cochin, AP-HP, F-75014 Paris, France; federico.bernabei89@gmail.com (F.B.); pierreraphaelrothschild@hotmail.com (P.-R.R.); 3 Ocular Oncology Service, Department of Surgical Oncology, Fondazione IRCCS Istituto Nazionale dei Tumouri, 20133 Milan, Italy; martina.angi@istitutotumouri.mi.it; 4 Ophthalmology Unit, S.Orsola-Malpighi University Hospital, University of Bologna, 40138 Bologna, Italy; marco.pellegrini@hotmail.it; 5 Medical Oncology Department, Santa Maria della Scaletta Hospital, 40026 Imola, Italy; a.maestri@ausl.imola.bo.it; 6 Department of Corneal and External Eye Diseases, St Paul’s Eye Unit, Royal Liverpool University Hospital, Liverpool L7 8XP, UK; vito.romano@gmail.com; 7 Department of Eye and Vision Science, University of Liverpool, Liverpool L7 8XP, UK; 8 Centre de Recherche des Cordeliers, Université de Paris, INSERM, UMR_1138, F-75006 Paris, France

**Keywords:** ocular tumours, radiotherapy, anti-cancer agents, ocular surface, complications, dry eye, choroidal melanoma, uveal melanoma, ocular surface squamous neoplasia

## Abstract

**Simple Summary:**

The ocular surface represents a finely regulated system that allows the protection of the eye. It can be affected by therapies used for the treatment of various intraocular tumours, particularly conjunctival cancers and uveal melanoma. In these conditions, treatments are chosen according to the characteristics of the lesion, and include a combination of selective surgery, anticancer eye drops, and/or radiotherapy delivered through different mechanisms. Possible side effects affecting the ocular surface range from transient dry eye or keratitis up to more severe complications such as corneal melting and perforation. These complications deserve careful evaluation for the risk of permanent sight-threatening sequelae. Physicians involved in the management of patients affected by ocular tumours should be aware of this risk in order to reach an early diagnosis and promptly set up an adequate treatment. The present review summarizes acute and chronic complications affecting the ocular surface following different therapies for the treatment of conjunctival cancers and uveal melanoma, and also reports clinical cases of representative patients who experienced these complications.

**Abstract:**

The ocular surface represents a finely regulated system that allows the protection of the eye. It is particularly susceptible to different treatments for intraocular tumours, such as uveal melanoma and conjunctival cancers. Traditionally, the management of ocular tumours depends on the characteristics of the lesion, and is based on a combination of selective surgery, topical chemotherapy, and/or radiotherapy delivered through different mechanisms (e.g., charged-particle radiotherapy or brachytherapy). Possible complications involving the ocular surface range from transient dry eye disease or keratitis up to corneal melting and perforation, which in any case deserve careful evaluation for the risk of permanent sigh-threatening complications. Clinicians involved in the management of these patients must be aware of this risk, in order to reach an early diagnosis and promptly set up an adequate treatment. The present review of the literature will summarize acute and chronic complications affecting the ocular surface following different therapies for the treatment of ocular tumours.

## 1. Introduction

Ocular tumours encompass a wide spectrum of disorders that represent important sight- and life-threatening conditions [1]. The treatments used for uveal and conjunctival tumours can lead to ocular surface complications which if not recognized and treated properly can lead to serious consequences. Uveal melanoma (UM) is the most frequent primary intraocular malignancy in adults. Most cases of UMs are represented by choroidal melanoma, while the remaining lesions arise from the ciliary body and the iris [2,3,4,5,6]. The most common conjunctival cancers are represented by melanocytic lesions and ocular surface squamous neoplasia (OSSN) [7]. Melanocytic tumours include conjunctival naevi, complexion-associated melanosis, ‘primary acquired melanosis’ (PAM), and invasive melanoma [8,9,10,11,12,13]. OSSN includes a broad spectrum of ocular surface neoplastic changes ranging from non-invasive squamous conjunctival intra-epithelial neoplasia to invasive squamous cell carcinoma [14,15].

Historically, the management of ocular cancers has been surgical (e.g., enucleation or, in advanced cases, orbital exenteration), resulting in significant functional and phycological morbidity. The introduction of ocular radiotherapy (e.g., proton beam radiotherapy, brachytherapy, and stereotactic radiotherapy) and the development of targeted topical treatments (e.g., mitomycin C [MMC], 5-fluorouracil [5-FU], interferon [IFN] -α2b), that can be used either alone or as combined treatments [16,17], have increased the chances of preserving the anatomy and the function of the affected eye, while limiting the spread of the malignancy [1].

The ocular surface consists of the palpebral and bulbar conjunctival epithelium, the corneoscleral limbus, the corneal epithelium, and the tear film. It represents the interface between the functioning eye and the environment, and it is a finely regulated complex system that provides anatomic, physiological, and immunologic protection of the eye [18]. Both anticancer drugs and radiotherapy can lead to ocular surface morbidities, ranging from transient dry eye disease or keratitis up to corneal melting and perforation [19]. Awareness of the potential ocular surface side effects of these treatments is crucial for early diagnosis, thus avoiding long-term severe complications. Thus, the aim of this review is to summarize the acute and chronic, relevant ocular surface complications that can occur following different therapies for the treatment of ocular tumours.

## 2. Mitomycin C

Mitomycin C is an alkylating agent isolated from *Streptomyces caespitosus* that inhibits cell replication by blocking DNA synthesis. Although MMC is toxic for both proliferating and non-proliferating cells, its action is more pronounced in hypoxic conditions and in cells with a higher mitotic rate, creating a certain level of selectivity [15]. Mitomycin C is used in concentrations of 0.02%–0.04% depending on the severity of the disease to be treated. It is generally administered four times a day for seven days followed by 1 or 2 weeks of suspension, commonly for four cycles. However, some authors have reported 2 weeks of continuous treatment followed by 2 weeks of interruption, repeated twice [19,20,21,22,23,24,25,26,27].

Topical MMC is commonly used for the treatment of both melanocytic tumours and OSSN, for either preoperative tumour debulking or postoperative prevention of recurrence [28,29,30,31,32].

The ocular surface complications reported following the use of MMC in patients affected by OSSN are presented in Table 1.

**Figure 1:**
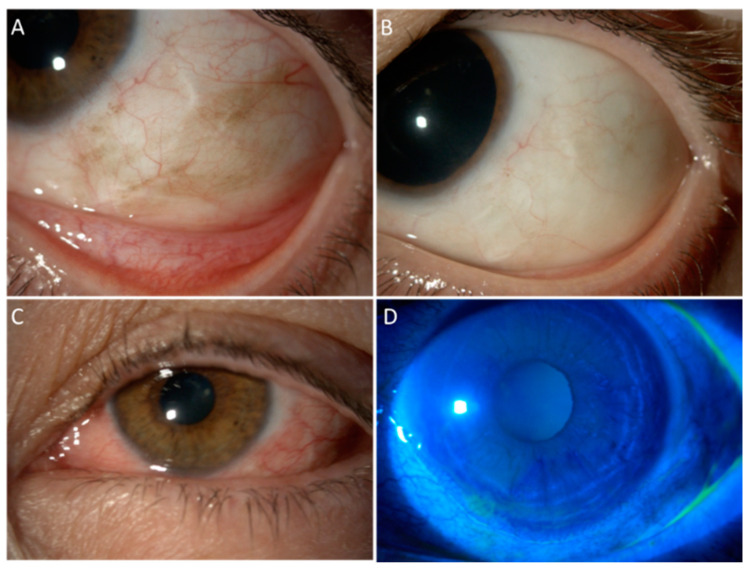
An example of MMC toxicity that successfully resolved with the treatment. A 54-year-old female suffering from biopsy-proven enlarging PAM with moderate atypia in the left eye, which had already been treated with surgical excision and conjunctival graft 8 years earlier for localized PAM with severe atypia. Part (**A**): Slit lamp picture at baseline, with tumour recurrence surrounding the paralimbal scar of the previous surgery. Part (**B**): Complete disappearance of pigmented cells 2 years after four courses of MMC 0.02% (one drop QID for 7 days). Part (**C**): Allergic reaction to MMC starting from the second cycle of treatment, presenting with lid edema, conjunctival swelling, epiphora, and photophobia. Part (**D**): Fluorescein staining revealed a peripheral superficial corneal epithelial defect. The allergic reaction has been managed with cold compresses, artificial tears, vitamin A ointment, and suspension of MMC. Once the epithelium was completely healed, MMC has been started again in association with weak steroid eye drops and close surveillance.

Although the current literature shows contrasting data regarding MMC side effects, an allergic reaction is the most frequently reported ones. Since this complication typically occurs during the second or third cycle of treatment, a delayed hypersensitivity reaction could be the causative mechanism [26]. The allergy typically settles rapidly on cessation of treatment. Corneal epithelial toxicity is another common complication following topical MMC treatment. Studies have reported corneal epitheliopathy in up to 50% of eyes with corneal epithelial defects reported in up to 18% [24,27]. However, other studies have reported the complete absence of corneal toxicity [22,26]. This difference does not seem to be related to drug concentration, but it could be explained by the use of topical lubrication used during the treatment cycle [22]. Epiphora due to punctal stenosis is another common complication, occurring in up to 17% of treated eyes [19,26]. Although sometimes this complication may be successfully resolved by simply irrigating the nasolacrimal drainage system [25,27], other cases may require punctoplasty or even dacryocystorhinostomy [19,26]. In order to avoid this complication, some authors suggest the insertion of punctal plugs prior to initiating the treatment with topical MMC [33]. Less commonly, MMC may cause lid toxicity resulting in lid edema, ectropion or ptosis due to levator disinsertion [24,25,26,27]. Furthermore, limbal stem cell deficiency (LSCD) has also been reported following topical MMC treatment [34]. It has been suggested that some corneal complications are secondary to LSCD, which may be underdiagnosed without an impression cytologic analysis [35]. A study using topical MMC as a primary treatment for PAM with atypia reported conjunctival hyperemia in all the 12 treated patients (100%), lid inflammation in two (17%), corneal epitheliopathy in two patients (17%), corneal epithelial defect in one patient (8%), and severe keratopathy in one patient (8%) [36]. Another study employing topical MMC for residual epithelial disease or as an adjuvant to excision and cryotherapy reported transient keratoconjunctivitis in all the 16 treated patients (100%), corneal neovascularization in two patients (13%), and corneal abrasion with scar formation in one patient (7%) [37]. Finally, a report on adjuvant MMC after surgery reported conjunctival hyperemia in 13 patients (87%), epiphora in 10 patients (67%), LSCD in four patients (27%), epithelial defect in one patient (7%), and lid edema in one patient (7%). Figure 1 shows the prompt management of MMC toxicity in a patient affected by relapsing PAM.

**Figure 2:**
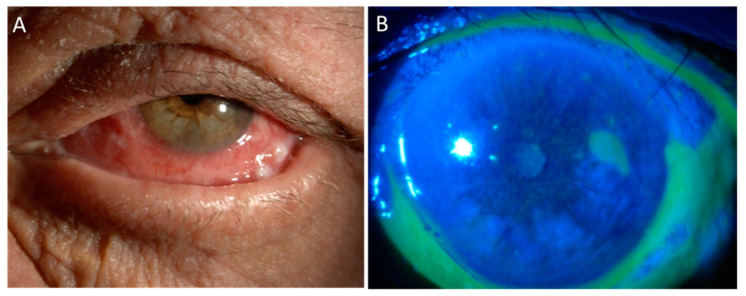
Toxic blepharoconjunctivitis following MMC. An 80 year-old man affected by relapsing squamous cell carcinoma of the lower fornix in his left eye, who had been treated elsewhere by repeated surgical excisions, presented to our Center after 15 days of continuous treatment with MMC 0.04% QID, with Part (**A**) severe orbital swelling, erythematous-desquamative blepharitis, and Part (**B**) corneal epithelial defect and diffuse conjunctival melting with pseudomembranes.

Figure 2 shows toxic blepharoconjunctivitis following MMC after surgical removal of conjuctival squamous carcinoma.

Preventive strategies should be adopted in order to minimize the side effects related to the use of MMC. In particular, the regular use of artificial tears during the day and vitamin A ointment at night are beneficial and should be prescribed for the entire duration of the treatment, as well as in the subsequent months [38]. Applying cold compresses over the eyelids can also relieve symptoms and reduce blepharitis. In addition, the topical administration of weak steroids, such as fluorometholone 0.1% or loteprednol etabonate 0.5% that are less likely to induce the onset of side effects such as cataract or glaucoma, allows the reduction of local inflammation [39,40]. These eye drops can be used during the treatment period and then tapered in the following weeks, ensuring that the further cycle of treatment is not started unless the eye is quiet. As a general rule, it is highly encouraged to perform slit lamp examination with fluorescein staining before starting a new cycle of treatment to assess the integrity of the ocular surface epithelium. Moreover, for correct timing of adjunctive topical chemotherapy, it is advisable to wait for the complete healing of surgical wound before starting the treatment.

Advising the patient that the side effects may increase with the number of cycles and to promptly report the onset of symptoms such as epiphora, photophobia, and orbital swelling is important for the early detection and management of potentially severe complications, such as corneal ulcers.

It should be noted that, as demonstrated in studies related to glaucoma surgery, the use of MMC reduces the density of conjunctival goblet cells [41]. These cells are responsible for the production of mucins contained in the tear film and their deficiency represents one of the mechanisms underlying the vicious cycle of dry eye disease [42]. This detrimental effect could contribute to the ocular surface alterations occurring in patients following the use of MMC.

## 3. 5-Fluorouracil

The 5-fluorouracil is a pyrimidine analog that inhibits the enzyme thymidylate synthetase, and thus impairs DNA and RNA synthesis. Since the amount of nucleic acid synthesis is higher in rapidly proliferating cells, the drug has a relative selectivity for tumour cells [43]. In addition, 5-fluorouracil at 1% concentrations is generally used four times a day for 1 or 2 weeks followed by 2 to 3 weeks of suspension, although some authors report a 4-week course of continuous treatment [44,45].

### Ocular Surface Squamous Neoplasia

The ocular surface complications reported following the use of 5-FU for OSSN are presented in Table 2 [19,27,44,45,46,47,48].

Although studies in the literature report conflicting results regarding corneal epithelial toxicity, 5-FU seems to be better tolerated than MMC [46,47,49]. Rudkin et al. reported an incidence of epithelial defects in 8% of eyes treated with 5-FU compared to 18% of eyes treated with MMC [8]. Midena et al. showed that superficial keratitis occurred in all the eight treated eyes, but the complication was successfully managed in 1 week with tear substitutes [44]. A single case of corneal stromal melting with visual impairment was reported in another study [27]. Lid erythema and inflammation are among the most com-mon complications following the topical 5-FU treatment, occurring in up to 62% of cases [30]. These complications are attributed to spillage of the eye drops onto the eyelid skin [46]. Some authors suggested to apply an ophthalmic ointment on the inferior eyelid prior to 5FU administration [45]. In addition, it is advisable to instruct the patient on the correct handling of the drug, encouraging the instillation of eye drops by a trained member of the family in order to minimize the risk of spilling. Epiphora may be a common complication of 5-FU [46], with 49% of eyes treated with 5-FU presenting this symptom after 1 month in a randomized controlled trial comparing 5-FU with placebo [46]. Although several studies reported the occurrence of epiphora due to stenosis of the lacrimal punctum, it must be taken into account that epiphora may also occur due to other causes such as reflex tearing due to ocular surface irritation from local chemotherapy.

## 4. Local Immunotherapy

IFNs are immunomodulatory cytokines released by human leukocytes in response to tumours or viral infections. IFN-α2b is a recombinant form of IFN-α which increases the host recognition and targeting of tumour cells by upregulating antigen presentation to T-lymphocytes [48]. The drug can be used as either an eye drop or as a subconjunctival/perilesional injection. Topical IFNα-2b is usually prescribed four times a day without interruption until 1—2 months after clinical resolution of the lesion. The most commonly used concentration is 1 million IU/mL. No significant differences between 1 and 3 million IU/mL concentration were found in a comparative study [50].

IFN-α2b is well tolerated and is associated with less ocular side effects than both MMC and 5-FU. However, IFN drops need to be kept refrigerated. This aspect, together with the higher cost as compared to MMC or 5FU, make it less widely used.

The most common ocular surface complication is conjunctival hyperemia, occurring in 4–13% of treated patients [23,51,52,53]. No corneal complications have been reported except for superficial punctate keratitis in three patients, an epithelial defect in one patient, and epithelial microcyst formation in one patient [52,53,54].

Intralesional injection of INF-α is a promising treatment for conjunctival lymphoma, which may be considered an alternative to radiotherapy. Blasi et al. reported local control rates of 85% after a median follow-up of 65 months. All patients reported transient conjunctival chemosis at the site of injections and a flu-like syndrome lasting 1 to 5 h [55].

## 5. Cryotherapy

Cryotherapy is a procedure usually applied to the surrounding areas of flat pigmentation at the time of conjunctival tumours surgery [56,57,58]. This procedure works by a combination of mechanical cell injury, ischemic necrosis, and immunologic response to released tumour antigens [59,60]. Although effective, cryotherapy can be associated with complications that include tarsal floppiness, ptosis, symblepharon, anterior uveitis, hypotony, scleral thinning, and melting [56,57,58,59,60]. Shields et al. suggested that lifting of the conjunctiva from the globe could help decrease the chances of ocular complications [57].

## 6. Radiotherapy for Uveal Melanoma

Small and medium sized UMs can often be treated with eye-conserving radiotherapy. This can be administered in the form of proton beam therapy (PBR), brachytherapy (BT) or stereotactic radiotherapy [61,62,63,64,65].

### 6.1. Proton Beam Radiotherapy

Proton beam radiotherapy is an effective treatment for UM of any size and location. It results in local control of the disease in more than 95% of cases and in a relatively high rate of eye preservation [66,67,68,69,70,71]. Side effects of PBR depend mainly on the size and location of the tumour [72,73,74,75,76,77,78]. Radiation keratopathy represents a possible complication after PBR occurring in 1–11.5% of cases [66,68,78,79]. Decreased corneal sensitivity up to complete anesthesia is a typical early sign of radiation keratopathy [80]. The loss of corneal sensory innervation leads to the impairment of both protective reflexes and epitheliotropic neuromodulators resulting in painless central or marginal corneal ulceration [81]. This complication is also related to the LSCD that occurs in approximately 30% of patients undergoing total anterior segment irradiation [62,77,82]. LSCD can be avoided by harvesting limbal corneal tissue before PBR and transplanting it after irradiation [83,84].

**Figure 3:**
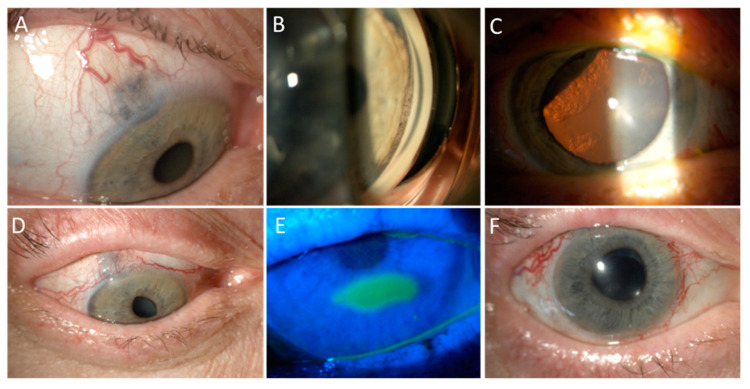
Anterior surface toxicity of PBR. Part (**A**): A 61 year-old lady presented with a large cilio-choroidal melanoma with scleral and iris invasion in the right eye, which was her only eye, as the left one had been previously enucleated due to a trauma. Part (**B**): Circumferential invasion of the iris angle by pigmented cells was detected on gonioscopy. Part (**C**): Sectorial cataract was present due to lens infiltration by the melanoma, as well as inferior exudative retinal detachment. The patient was treated with PBR sectorial irradiation of the ciliary body, anterior choroid, and whole iris. Harvesting of limbal stem cell was not performed due to the extraocular extension. Part (**D**): Three months after treatment, the patient developed madarosis and scarring of the superior eyelid and diffuse punctate keratitis that was managed with the regular use of artificial tears and vitamin A ointment in association with atropine and unpreserved mild steroids. Part (**E**): Eight months after treatment, a neurotrophic keratopathy developed and was treated with gas-permeable contact lenses and hourly tear substitutes. Part (**F**): Two years after PBR, the tumour has regressed to a flat scar. The patient has undergone cataract surgery and anti-vascular endothelial growth factor injections, vitrectomy, and endolaser for neovascular glaucoma due to ischemic retinopathy, with a residual visual acuity of 20/200 due to radiation maculopathy.

Scleral necrosis represents an uncommon complication after PBR, that can lead to perforation in the most severe cases [85]. Risk factors include tumour thickness higher than 6 mm and the involvement of the ciliary body [85]. In severe cases this complication may require kereatoplasty, possibly using a lamellar flap from the same eye [85]. The upper eyelid could also be damaged by PBR. The transpalpebral procedure has been proposed in order to avoid collateral damage to the upper eyelid margin without increasing the risk of failure of local tumour control [86]. In addition, the lacrimal drainage system could also be affected with the development of canaliculitis or punctal obstruction [73]. Other complications reported following PBP include pseudophakic bullous keratopathy (2%) and dry eye disease (6%) [84,87,88,89]. However, most studies reported no impairment of the ocular surface [67,75,90,91,92,93,94,95,96,97,98]. Figure 3 shows the management of toxicity in a patient affected by large cilio-choroidal melanoma with diffuse iris invasion, treated with PBR to the entire anterior segment.

### 6.2. Brachytherapy

The advantage of BT for the treatment of posterior UMs is the distribution of less radiation to the structures of the anterior segment [61,99,100,101,102]. Compared to charged particle radiotherapy, BT results in a lower incidence of anterior segment complications [103]. In particular, BT could lead to complications to the sclera, the conjunctiva, and more rarely the cornea [104]. In a series of 239 patients treated with high intensity Iodine-125 plaques (minimum tumour dose of 70 Gy in 4 days), keratitis was present in 34% of them, epiphora was present in 23.5%, and dry eye in 8.1% [105]. In another series of 136 cases treated with Iodine-125 plaques, 2.8% of patients presented with keratitis after 2 years [106]. Finger et al. reported the presence of dry eye in 15% of patients treated with Palladium-103 plaque for different types of UMs [82]. Ruthenium plaques rarely give rise to anterior segment complications. However, conjunctival dehiscence and scleral necrosis have been reported, particularly when conjunctival closure is inadequate in the area of muscle disinsertion. In these cases, the repair of conjunctival dehiscence must be promptly performed. Subsequently, a conservative treatment with intensive lubrication and corticosteroids or punctum plugs insertion may be required to reduce local inflammation and improve tear film quality [107]. Scleral necrosis should be promptly recognized in order to start immediately the adequate treatment to avoid more serious consequences. More severe cases may require a partial-thickness scleral patch graft that could be obtained from donor eyes [85]. Other studies reported no relevant alterations of the ocular surface after brachytherapy Iodine-125, Palladium-103, Ruthenium-106, and Strontium-90 [101,102,108,109,110,111,112,113,114,115,116,117,118,119,120,121,122,123,124,125,126,127,128].

### 6.3. Stereotactic Radiotherapy

Stereotactic radiation therapy is a precise radiotherapy technique utilizing photon beams. This technique is able to apply high doses of radiation in one or more fractions to a well-defined volume [129,130,131]. Stereotactic irradiation therapy performed using a linear accelerator (LINAC) device, Gamma-Knife and Cyber-Knife system, represents a feasible procedure for the management of UM, especially in the absence of PBR facilities [131,132,133,134,135,136,137].

A recent study reported blepharitis and long-lasting corneal epithelial defects in 16% and 15% of patients treated with Gamma-Knife radiosurgery for UMs, respectively [138]. Dunavoelgyi et al. reported corneal epithelial defects as the most common impairment of the ocular surface after LINAC treatment. In particular, corneal epithelial defects occurred in the first 3 months after therapy administration in 21% of patients, and this percentage remained stable for 5 years [139]. In a series of 158 patients treated with linear accelerator-based stereotactic fractionated, several acute side effects were reported: Blepharoconjunctivitis (5%), corneal epithelial defects (3%), epitheliolysis (5%), and madarosis (6%). These complications were more common if the tumour was in close proximity to the anterior segment. No long-term side effects were reported [135]. Another study reported that blepharitis and conjunctivitis occurred in 19% of treated patients as early complications [134]. In another series of patients treated with LINAC with a dose range of 37.5–131.25 Gy, one patient received enucleation due to a recurrent painful corneal ulcer 2 years after therapy [140]. Other authors reported no significant ocular surface complications after the procedure [134,141,142,143,144,145]. Cyber-Knife is a light-weight LINAC-based radiosurgery system that can also be used for the treatment of UMs [146]. To date, no major impairment of the ocular surface has been reported after treatment with the Cyber-Knife system, but there is still very limited literature on this technique [146,147,148].

## 7. Radiotherapy for Ocular Surface Tumours

### 7.1. Ocular Surface Squamous Neoplasia

In selected cases of invasive ocular surface tumours, brachytherapy or proton-beam radiotherapy may be used after resection to avoid recurrences as an alternative to enucleation [149,150,151]. In a series of 15 patients with scleral or intraocular invasive OSSN treated with Iodine-125 brachytherapy, Arepalli et al. reported the development of epithelial defect and corneal edema in 27% and 20% of treated patients, respectively. In one patient, the persistent epithelial defect caused chronic ocular irritation that required enucleation [149]. In another series of patients treated with Iodine-125, corneal ulceration and LSCD were reported in 45% and 9% of patients, respectively [152]. Another study using strontium-90 brachytherapy reported less ocular surface toxicity, with only three cases of scleral ulcer out of 123 treated patients [153]. Ocular morbidities, in particular scleral necrosis, are more frequent and generally more severe if radiotherapy is performed in the presence of a conjunctival defect, hence this should preferably be avoided. In addition, scleral melt is more frequent after a repeated radiation treatment on the same area of sclera.

**Figure 4:**
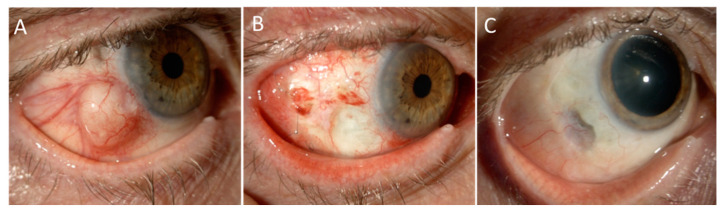
Ruthenium brachytheraphy for invasive squamous cell carcinoma of the conjunctiva. Part (**A**): A 71 year-old man affected by invasive squamous cell carcinoma of the conjunctiva in the right eye that aggressively recurred shortly after a partial excision with scleral lamellectomy and cryotherapy done at a local hospital. In the attempt to save the globe, he was treated with surgical excision, Ruthenium-106 brachitherapy, and ocular surface reconstruction with amniotic membrane graft. Part (**B**): One-month after surgery, the amniotic membrane had been completely reabsorbed and the patient was treated with two courses of adjuvant 5-FU and intensive topical lubrification and soft steroids with slow tapering. Part (**C**): Two years after treatment, the eye is preserved with a 20/20 vision with no signs of recurrence. Note the scleral thinning, well covered by tenon and conjunctiva, in the area of previous full-thickness tumour invasion. This area is being monitored by means of anterior segment optical coherence tomography to exclude its evolution. In such case, a wide scleral patch would be indicated.

In specific cases of invasive disease, brachytherapy could be used as a salvage treatment after surgical excision. In these cases, it may be necessary to perform brachytherapy even if the conjunctival healing is not achieved, and the use of amniotic membrane, intensive lubrication, and careful control of inflammation are essential to prevent scleral necrosis. A representative case treated according to this technique is presented in Figure 4.

A careful evaluation before starting the treatment is mandatory. The mechanisms underlying these alterations remain unclear and future studies are needed to address this issue. However, several factors can lead to an impairment of the ocular surface system. In particular, a direct cytotoxic effect can damage the corneal tissue but also lacrimal and/or meibomian glands. Furthermore, the damage of the sub-basal nerve plexus can lead to a reduced nervous trophism of the cornea, which determines the onset of neurotrophic keratopathy [149,150,152,153].

It must be emphasized that the location of the tumour and the site of positioning of the plaque is crucial for the development of subsequent complications. In particular, the treatment of a lesion at the corneal level will lead to complications such as persistent epithelial defect or corneal ulceration. Conversely, the treatment at the conjunctival level will mainly lead to complications such as dry eye, symblepharon or scleral necrosis.

### 7.2. Conjunctival Melanoma

Plaque brachytherapy may be used as an adjuvant treatment for conjunctival melanoma with margins tested positive for deep corneoscleral invasion [152,153]. Brachytherapy with Iodine-125 or Ruthenium-106 have been extensively used [152,154,155]. Karim et al. treated 19 patients with Iodine-125 and reported corneal ulceration in six of them (32%) [154]. Another study used Ruthenium-106 as a primary treatment in 40 patients, and as salvage therapy after surgery in 36 patients, reporting keratopathy in 12% of patients, trichiasis in 12% of patients, symblepharon in 9% of patients, and ptosis in 3% of patients [155]. Proton beam radiotherapy has been proposed as an alternative to exenteration in cases of extensive palpebral, forniceal or caruncular involvement [156]. Wuestemeyer et al. treated 20 patients of complicated conjunctival melanoma in unfavourable sites not eligible for brachytherapy: Ocular surface complications included dry eye disease (95% of patients) and LSCD (20%) [156].

### 7.3. Conjunctival Lymphoma

External beam radiotherapy represents an effective treatment for low-grade conjunctival lymphoma [157]. Radiotherapy alone, with a dose range between 20 and 30 Gy, resulted in a 5-year local control rate of 89%-100% [157]. The most common complication of the procedure is dry eye disease, with a reported incidence ranging from 14% to 45% [158,159,160,161]. In addition, a study reported keratitis and ulceration in 9% and 6% of patients, respectively. Another study reported a case of corneal perforation that required enucleation [159]. The largest and most recent study included 121 patients with conjunctival lymphoma, and reported the occurrence of dry eye in 27% of patients, tearing in 6% and eye pain in 5% [158]. Brachytherapy with strontium-90-yttrium-90 applicators has been proposed as an alternative to external beam radiotherapy in order to reduce the irradiation of orbital and intraocular structures. A study that included 10 patients treated according to this technique showed a local control rate of 77% after a median follow-up of 6.5 years. Ocular surface complications included conjunctivitis in 85% of patients and keratitis in 69% [162]. However, the availability of this radioisotope is limited worldwide.

## 8. Management of Ocular Surface Side Effects

Some treatments for ocular tumours can lead to poor ocular surface healing capacity. In certain high-risk patients, serial follow-up visits are recommended not only to monitor possible recurrences, but also to promptly diagnose and treat ocular surface diseases, thus preventing further complications. Punctate keratopathy needs aggressive lubrication: Tear replacement therapy with non-preserved tear substitutes and ointments facilitates epithelial wound healing. Recurrent corneal epithelial breakdown can be treated with bandage contact lens and punctal occlusion. Persistent epithelial defects can be treated with serum eye drops, bandage contact lens or also nerve growth factor in the case of reduced/absent corneal sensitivity [163]. Topical antibiotics can be used for a limited duration for the prophylaxis of infection. In the case of epithelial defect unresponsive to medical therapy, tarsorraphy, amniotic membrane transplantation or conjunctival flap can be used to protect or reconstruct the ocular surface. Limbal stem cell deficiency should be suspected in the presence of corneal neovascularization and investigated by means of conjunctival impression cytology, if present, limbal transplantation may be indicated. Prolonged and intense ocular surface inflammation can be managed by steroids or other anti-inflammatory agents when available (e.g., cyclosporine or lifitegrast).

Eyelid inflammation such as blepharitis or meibomitis and periorbital skin involvement can be acutely controlled with topical corticosteroids and antibiotic therapy.

In patients with lid defects, reconstructive surgery is necessary to address functional or aesthetic deficits. In the case of mild lagophthalmos, artificial tears can be administered frequently in order to improve the patient’s tear film. Moreover, eyelid taping at night offers additional ocular surface protection. In more severe cases, tarsorraphy or gold weight implantation are required.

## 9. Conclusions

Although recent advances in treatments have made therapeutic strategies increasingly targeted and personalized, both anticancer drugs and radiation therapy can lead to an impairment of ocular surface structures. This results in a wide spectrum of clinical pictures which deserves a careful evaluation and an appropriate treatment, in order to preserve the visual function as well as the quality of life of these patients. Their early recognition is crucial in order to promptly set up an adequate treatment able to avoid permanent sight-threatening complications.

It should be noted that the incidence rates of the complications reported in the literature are rarely adjusted for all risk factors and cofactors that could determine their onset. This issue represents a limitation of the data reported herein that should be taken into consideration by the reader. Another important limitation of this review is that the primary purpose of the studies in the literature is to evaluate the outcome of the therapies rather than the related complications. It is possible that the slightest alterations that occur at the level of the ocular surface, such as dry eyes or allergic reactions, are overlooked. This could explain the great difference that exists in the frequencies reported across the different studies.

## Figures and Tables

**Table 1:** Ocular surface complications of topical mitomycin C for ocular surface squamous neoplasia.

Study	Number of Patients	MMC Concentration	Allergy	Corneal Epitheliopathy	Epithelial Defect	Epiphora	Lid Inflammation	Ectropion	Ptosis
Bahrami 2013 [19]	64	0.04%	28%	0%	0%	17%	0%	0%	0%
Ballalai 2009 [21]	23	0.02%	0%	0%	17%	0%	0%	0%	0%
Birkholz 2011 [22]	17	0.02%	0%	0%	0%	0%	0%	0%	0%
Blasi 2018 [23]	16	0.02%	13%	13%	0%	0%	0%	0%	0%
Daniell 2002 [24]	20	0.02-0.04%	0%	50%	NS	0%	10%	0%	0%
Gupta 2010 [25]	91	0.04%	23%	0%	2%	15%	0%	0%	1%
Khong 2006 [26]	100	0.04%	34%	0%	0%	17%	1%	0%	1%
Rudkin 2014 [27]	39	0.04%	18%	23%	18%	5%	0%	3%	0%

MMC: Mitomycin c.

**Table 2:** Ocular surface complications of 5-fluorouracil for ocular surface squamous neoplasia.

Study	Number of Patients	Corneal Epitheliopathy	Epithelial Defect	Epiphora	Lid Inflammation	Ectropion
Bahrami 2013 [19]	89	6%	1%	10%	62%	1%
Gichuhi 2016 [46]	47	0%	0%	49%	14%	0%
Joag 2016 [47]	44	0%	0%	10%	2%	0%
Midena 2000 [44]	8	100%	0%	0%	0%	0%
Parrozzani 2016 [45]	41	28%	0%	0%	8%	0%
Rudkin 2014 [27]	12	0%	8%	0%	42%	0%
Venkateswaran 2018 [48]	54	7%	0%	22%	9%	0%
